# Food access, domestic environments, and dietary quality of low-middle income Chilean children during the COVID-19 pandemic

**DOI:** 10.3389/fpubh.2023.1164357

**Published:** 2023-06-20

**Authors:** Isabel Pemjean, Fernanda Mediano, Pedro Ferrer, María Luisa Garmendia, Camila Corvalán

**Affiliations:** ^1^Doctoral Program in Public Health, School of Public Health, University of Chile, Santiago, Chile; ^2^Carolina Population Center, University of North Carolina, Chapel Hill, NC, United States; ^3^Center for Research in Food Environments and Prevention of Nutrition-Related Diseases (CIAPEC), Institute of Nutrition and Food Technology, University of Chile, Santiago, Chile

**Keywords:** food environments, domestic food environment, food access, dietary quality, children, Chile

## Abstract

**Introduction:**

Food access is associated with dietary quality; however, people living in similar physical environments can have different food access profiles. Domestic environments may also influence how food access relates to dietary quality. We studied food access profiles of 999 low-middle income Chilean families with children during the COVID-19 lockdown and how these profiles relate to dietary quality; secondarily, we also explore the role of the domestic environment in this relationship.

**Materials and methods:**

Participants of two longitudinal studies conducted in the southeast of Santiago, Chile, answered online surveys at the beginning and end of the COVID-19 pandemic lockdown. Food access profiles were developed by a latent class analysis considering food outlets and government food transfers. Children's dietary quality was estimated by self-reported compliance with the Chilean Dietary Guidelines of Americans (DGA) and daily ultra-processed food (UPF) consumption. Logistic and linear regressions were used to assess the association between food access profiles and dietary quality. Domestic environment data (i.e., the sex of the person who buys food and cooks, meal frequency, cooking skills, etc.) were incorporated in the models to assess their influence on the relationship between food access and dietary quality.

**Results:**

We have categorized three food access profiles: Classic (70.2%), Multiple (17.9%), and Supermarket-Restaurant (11.9%). Households led by women are concentrated in the Multiple profile, while families from higher income or education levels are focused on the Supermarket-Restaurant profile. On average, children presented poor dietary quality, with a high daily UPF consumption (median = 4.4; IQR: 3) and low compliance with national DGA recommendations (median = 1.2; IQR: 2). Except for the fish recommendation (OR = 1.77, 95% CI:1.00–3.12; *p*: 0.048 for the Supermarket-Restaurant profile), the food access profiles were poorly associated with children's dietary quality. However, further analyses showed that domestic environment variables related to routine and time use influenced the association between food access profiles and dietary quality.

**Conclusion:**

In a sample of low-middle income Chilean families, we identified three different food access profiles that presented a socioeconomic gradient; however, these profiles did not significantly explain children's dietary quality. Studies diving deeper into household dynamics might give us some clues on intra-household behaviors and roles that could be influencing how food access relates to dietary quality.

## 1. Introduction

Poor-quality diets are the leading risk factor for premature death globally, and improving diets could prevent one in five deaths (1). Difficulties in food acquisition (i.e., economic, physical, and social) (2) have been identified as one of the most critical variables concerning diet quality, particularly in low- and middle-income communities (3, 4). However, studies assessing the association between food access and specific health, or nutritional outcomes provide inconsistent results (5, 6). The lack of specificity in particular instruments and indicators (6, 7) may be responsible for the inability to differentiate between populations living in similar physical environments.

The food environment has been defined as “the interface that mediates people's food acquisition and consumption within the wider food system” (2). When considering the food environment, there are two main dimensions: external factors such as availability, prices, food properties, and marketing, as well as personal factors such as accessibility, affordability, convenience, and how desirable the food products are. For children and adolescents, this also includes their behaviors and those of their caregivers when procuring, preparing, and eating food (8). The domestic environment is a setting where personal and behavioral dimensions occur. It is the primary space where children socialize and learn about food tastes, preparation, and traditions (9). However, it is rarely explored (2, 10). Recent evidence suggests that domestic environment variables, such as gender (11), and intra-household relationships (12, 13), can reflect coping strategies (14, 15) that ultimately shape how food environments impact people's life (16).

In Chile, food insecurity is no longer an issue (17); however, excessive consumption of nutrients related to nutrition-related chronic diseases such as sugars, sodium, and saturated fats is a major concern (18). Evidence of dietary quality among children is particularly scarce but suggests large consumption of ultra-processed foods (19). Chile has a modern food system (20) characterized by an abundance of food, including the so-called ultra-processed foods (UPF) that have been related to a high risk of obesity and other chronic diseases (21). People live mostly in urban settings with a high concentration of market-based food sources, including many options to eat outside the home (22). Before the pandemic, supermarkets and markets were important food supply places in Latin-American countries (LAC) and Chile, with some peculiarities. Chile is one of the LAC countries with the highest expansion of supermarkets. Along with this growth, the sales of unhealthy processed foods have increased over the last decade (23). Supermarkets have replaced shopping in neighborhood grocery and convenience stores, which have now become places where people buy discretionary foods or ingredients to cook a single meal (24). Eating outside of the home or purchasing take-home foods from restaurants has also increased, particularly in high-income levels (24). In Santiago, the capital of Chile, open markets concentrate in low-middle-income sectors (25). Although they represent the main point of fruits, vegetables, and fish distribution (26), they currently offer all types of foods, including UPF. Chile also has strong social welfare and healthcare programs that consider the provision of free food through school and primary healthcare centers to groups with special nutritional needs, such as pregnant women, infants, and older adults.

In 2020, the country was hit by the COVID-19 pandemic, implementing rigorous confinement and lockdown measures, including curfews and school closures for the entire year. Food availability was not interrupted because of intense efforts to ensure food distribution and availability (27). Although open fairs were initially closed, they were quickly reopened. Additionally, agricultural production was declared an essential activity ensuring its continuity. However, food acquisition was hampered by restrictions on people's movement (20). To mitigate these effects, the government implemented food assistance measures: the school feeding program was replaced by the delivery of food boxes for in-home meal preparation, and a food box program targeted to low-income families was implemented at two points during the 1st year of the pandemic.

Thus, the current project aimed to describe food access profiles of low-middle income families participating in the longitudinal studies of the Center for Research in Food Environments and Prevention of Nutrition-Related Diseases (CIAPEC) conducted in the southeast area of Santiago, Chile, during the COVID-19 lockdown and how they relate to the dietary quality of children and adolescents during the same period; we secondarily explore how the domestic environment influenced this relationship.

## 2. Materials and methods

### 2.1. Study design

This was a longitudinal study. Food access and domestic food environment variables were collected from July to November 2020, (i.e., the first lockdown of Santiago), and dietary quality variables were measured in December 2020–February 2021, (i.e., lockdown opening) in households with children and adolescents participating in the CIAPEC cohorts. Data were primarily collected online (1582; 79.2%) and complemented with phone interviews (416; 20.8%).

### 2.2. Study participants

All families who participated in two longitudinal studies conducted in the Southeast area of Santiago in 2020 were invited to participate. The Growth and Obesity Cohort Study (GOCS) comprises children born in 2002–2003 recruited from public nursery schools in 2006. The inclusion criteria included being singleton term births, having birth weights between 2,500 and 4,500 g, and being free from conditions that affect growth (28). The Food Environment Chilean Cohort (FECHIC) includes children born in 2011–2012 and recruited in 2016 following the same recruitment procedures and inclusion criteria in the GOCS. According to the characterization of their neighborhoods of residence, households in both cohorts are considered low- and middle-income (29).

Only participants who continue to reside in the southeastern area of Santiago and who completed the sociodemographic and dietary sections of the surveys were included in this study. A sample of 999 households was obtained: 531 GOCS adolescents [17.6 years (SD 0.7), 54.8% girls] and 468 FECHIC children [8.8 years (SD 0.8), 51.9% girls]. Compared to the initial cohorts, participants did not differ significantly in age, sex, and nutritional status. However, maternal education was higher than in the initial cohorts, and more women participated only in the GOCS cohort.

This study was conducted following the Declaration of Helsinki, and the Ethics Committee of the Faculty of Medicine (University of Chile) approved his protocol.

### 2.3. Variables

Variables were defined as (1) outcomes: children's dietary quality; (2) predictors: food access profiles; (3) domestic environment variables; (a) food management; (b) COVID-19 pandemic routine modification (from now on, routine modification variables), and (4) sociodemographic covariables. The data collected related to the two weeks before the interview. In the case of FECHIC, the caregivers provided the data, while in the case of GOCS, the adolescents did it.

Dietary quality was measured by two variables: UPF consumption and accomplishment of the Chile dietary guidelines (DGA) (30). UPF consumption was measured with the NOVA score for the consumption of ultra-processed foods (31) that has been validated in Brazil (32) and in Chilean adolescents and young adults (under review data show good concordance with R24). The NOVA score is a 0 to 20-point scale: the consumption of each 20 UPF the previous day is counted as 1 point. DGA compliance is a 0 to 5-point scale where compliance with recommended consumption for each food group (fish, legumes, dairy, fruits and vegetables, and water) is counted as 1 point.

Food access profiles were estimated based on market-based food outlets (binary categorical variables of food purchase in the supermarket, open market, neighborhood grocery, larger food market, convenience store, pharmacy, bulk store, and restaurant), and government food transfers grouped into two binary categorical variables (received food support from the government before and during COVID-19). Given the urban nature of the study sample, we considered that own productions and harvested food did not apply as a food source in this context.

Sociodemographic variables included median household income according to Chilean minimum wage, head of household sex, mother's educational level, numerous households (more than three children), and sex and age of the child or adolescent.

We also explored the role of domestic environment variables: (1) food management: this set of variables included the sex of the main person in charge of buying and cooking food, with mastery of five or more cooking skills (boiling, steaming, frying, sautéing, grilling, baking/roasting, stewing, and microwaving) and the child meal frequency pattern (breakfast, lunch, and dinner). (2) Routine modification variables: This set of variables included increased hours dedicated to online work and housework, decreased hours dedicated to in-person work, and difficulties due to school closures in different areas. A more equitable gender distribution of food tasks (33, 34), the ability to prepare meals with a variety of cooking skills (35), and children having a meal frequency pattern have been described as protective factors for the child's quality diet (36). On the contrary, time constraints have been defined as risk factors for children's quality diet (37, 38). The COVID-19 lockdown measures (i.e., increased online work and school closures) raised household chores and stressed available time (39).

### 2.4. Statistical analysis

Quantitative variables were described as mean and standard deviation or as the median and interquartile range (if not normally distributed, assessed by the Shapiro–Wilk test) and categorical variables as number and percentage.

External food access profiles were elaborated with the latent class analysis (LCA). The LCA was chosen because the main food supply sites are considered a proxy for hidden socio-cultural variables (40). We incorporated the sociodemographic variables into the LCA model, so it reported the logistical/multinomial regression results between them and the food access profiles. This approach was preferred to estimating the model without covariates and then estimating their association with the covariates since the literature has shown that the latter produces downward biased estimates of the effects of the covariates (41). We estimated the degree of model's fit with one to three profiles (the model stopped converging at three profiles). Following the principle of parsimony and goodness-of-fit (Akaike Information Criteria and Bayesian Information Criteria), we selected the three latent class models with the following sociodemographic variables: median household income, head of household sex, and mother's educational level (all *p*-values <0.05). We assigned a name to each profile highlighting their food access differences using the item-response probabilities. Finally, households were assigned to a profile according to the posterior probability predictions (41, 42).

According to Gudicha et al. (43), a sample size of 999 is sufficient to obtain power over 95% in estimating three classes, even with a low association between the class and the indicator and unequal sizes between classes. This sample size would also allow us to find statistically significant small effect sizes assuming a power of 80% and an alpha error of 5% and also three predictors (44).

Associations between food access profiles and children's dietary quality were estimated using linear regression models for discrete variables and logistic regression models for categorical variables, adjusting for the sex and age of the child. To explore the effect of the domestic environment in these associations, we further adjusted the models by the domestic variables associated with the food access profiles. We incorporated the food management covariables first and then the routine modification variables. We compared the effects of the crude and adjusted regressions.

Associations between food access profiles and domestic food environment were estimated using multinomial regressions adjusted for the sex and age of the child. First, we estimated univariate models, and for each set of covariables (food management and routine modification), we retained those with a *p*-value of <0.05. Second, we estimated multivariable models for each set of covariables and retained those with a *p*-value <0.05.

For all analyses, interactions by the cohort study were tested and found non-significant; thus, analyses are combined for both cohorts.

All analyses were done with Stata v 16. All materials are available upon request to interested researchers.

## 3. Results

### 3.1. Descriptive results

Nine hundred and ninety-nine households participated in the study (Table 1). The median age of children was 13.5 years (IQR:9), with a similar number of girls and boys. Almost 2/3rds of the households (77.7%) received less than two minimum wages^1^ per month and 40.2% of the households received less than one. Women were the head of the household in almost half of the sample (47.2%), and they were also responsible for almost all food purchases (82.6%) and food preparation (92.3%).

**Table 1:** Main variables from 999 households in southeastern Santiago, Chile.

**Variable**	***n***	FECHIC^*^ ***n* (%)**	GOCS^**^ ***n* (%)**	**ALL** ***n* (%)**
**Sociodemographic variables**				
Sex of child/adolescent, female	999	243 (51.9)	291 (54.8)	534 (53.5)
Age of child/adolescent, median (IQR)	999	8.8 (0.8)	17.6 (0.7)	13.5 (4.5)
Median household income < 2 minimum wages	999	337 (72.0)	439 (82.7)	776 (77.7)
Female head of household	999	178 (38.0)	293 (55.2)	471 (47.2)
Mother completed higher education	999	148 (32.6)	104 (20.7)	252 (26.4)
≥3 children/adolescents in the household	999	90 (19.2)	79 (14.9)	169 (16.9)
**Predictor variables**				
Supermarket food shopping	999	325 (69.4)	369 (69.5)	694 (69.5)
Open market food shopping	999	302 (64.5)	378 (71.2)	680 (68.1)
Neighborhood grocery food shopping	999	276 (59.0)	314 (59.1)	590 (59.1)
Pharmacy food shopping	999	105 (22.4)	134 (25.2)	239 (23.9)
Food market shopping	999	47 (10.0)	46 (8.7)	93 (9.3)
Larger food market shopping (Vega/Valledor)	999	13 (2.8)	27 (5.1)	40 (4.0)
Bulk store food shopping	999	15 (3.2)	16 (3.0)	31 (3.1)
Restaurant food shopping	999	23 (4.9)	10 (1.9)	33 (3.3)
Convenience store food shopping	999	6 (1.3)	10 (1.9)	16 (1.6)
Government food support before COVID-19	999	285 (60.9)	371 (69.9)	656 (65.7)
Government food support during COVID-19	999	379 (81.0)	465 (87.6)	844 (84.5)
**Domestic environment variables**				
**Set of food management variables**				
Woman buys food	995	371 (79.4)	451 (85.4)	822 (82.6)
Woman prepares food	988	438 (94.2)	474 (90.6)	912 (92.3)
Child/adolescent has a meal frequency pattern	871	161 (39.4)	65 (14.1)	226 (26.0)
Master ≥5 culinary skills	940	157 (35.4)	177 (35.6)	334 (35.5)
**Set of routine modification variables**				
Increased domestic work hours, yes	984	286 (61.8)	249 (47.8)	535 (54.4)
Increased online work hours, yes	942	146 (32.9)	108 (21.7)	254 (27.0)
Reduction in working hours in person, yes	934	132 (29.8)	173 (35.2)	305 (32.7)
**Difficulty due to school closures**				
Making time for myself without the children	946	184 (40.5)	132 (26.8)	316 (33.4)
Having a schedule for school activities at home	965	183 (39.2)	163 (32.7)	346 (35.9)
Maintain communication with teachers	955	106 (22.9)	131 (26.6)	237 (24.8)
Monitor the child's school activities	965	193 (41.3)	114 (22.9)	307 (31.8)
Support the child's learning at home.	965	156 (33.4)	131 (26.3)	287 (29.7)
**Outcome variables**				
Daily intake of ultra-processed foods by the child/adolescent, median (IQR)	878	4.6 (2.2)	4.2 (2.1)	4.4 (2.2)
≥5 ultra-processed foods consumed daily by the child/adolescent	878	202 (48.9)	137 (42.4)	399 (45.4)
Number of DGA recommendations accomplished by the child/adolescent, median (IQR)	877	1.2 (1.0)	1.2 (1.1)	1.2 (1.0)
≥3 DGA recommendations accomplished by the child/adolescent	877	72 (11.7)	17 (10.8)	11 (10.6)

* FECHIC, The Food Environment Chilean Cohort.

** GOCS, The Growth and Obesity Cohort Study.

During the first COVID-19 lockdown, almost a third (32.7%) of the households decreased in-person work, with a slightly lower increase in online work (27.0%). Conversely, hours dedicated to domestic work increased in more than half of the households (54.4%). One-third of them experienced some difficulty associated with the closing of the schools either having time for themselves without the child (33.4%), keeping a schedule for school activities at home (35.9%), or controlling the child's homework at home (31.8%).

On average, children presented poor diet quality, with a high UPF consumption [median = 4.4; (IQR:3); 45.4% >5 UPFs per day]; and low compliance with national DGA recommendations [median = 1.2 (IQR:2); 11.4% ≥3 DGA].

Regarding the places of food acquisition during the lockdown, households bought their food mainly in supermarkets (69.5%) and open markets (68.1%) and slightly less in neighborhood grocery stores (59.1%). However, pharmacies (23.9%) are also noteworthy for their high prevalence. Almost two-thirds of the study families received food support from the government before the pandemic (65.7%), and this figure increased to 84.5% during the pandemic.

### 3.2. Food access profiles

**Figure 1:**
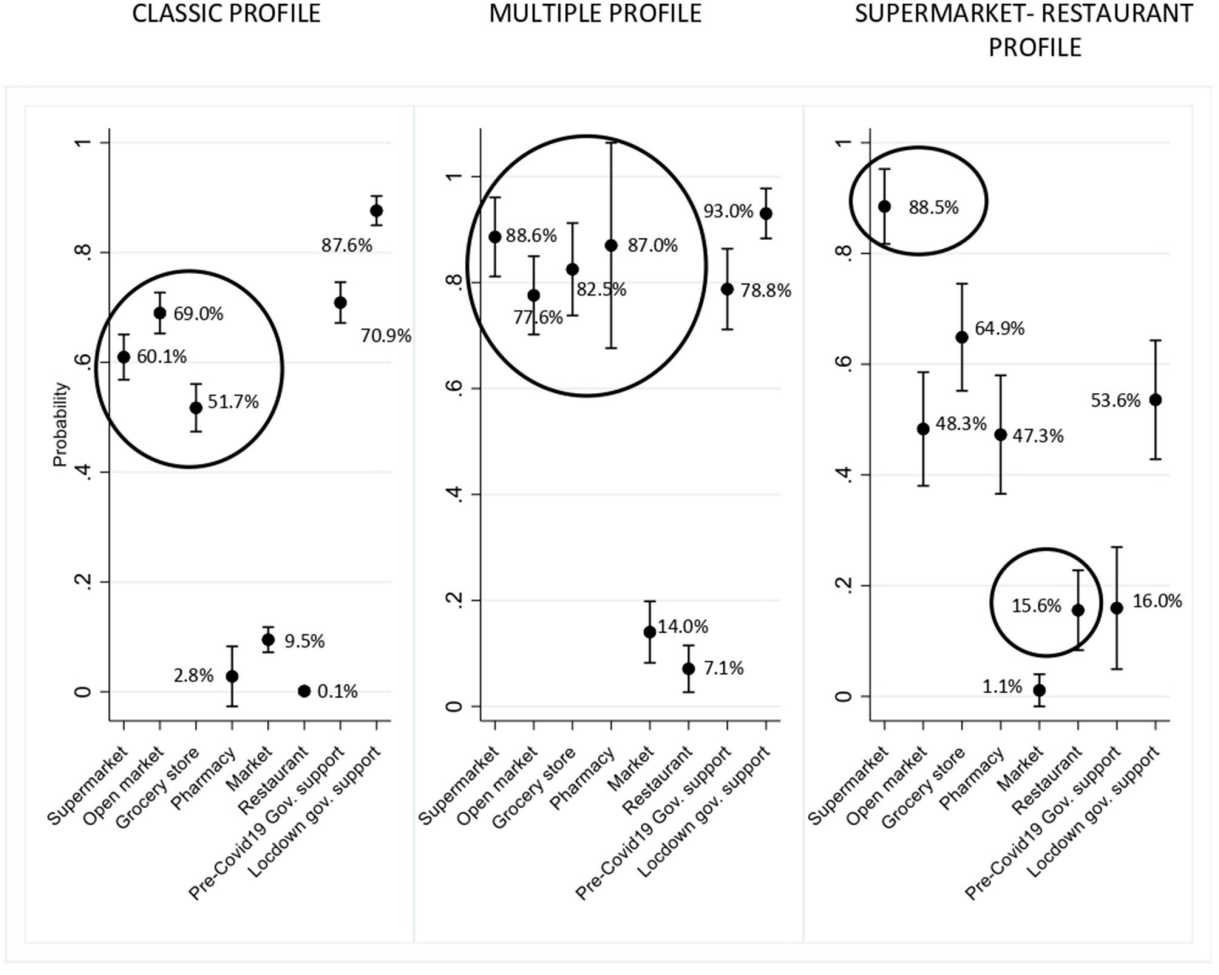
Food access profiles and main places of food access. 999 households in Southeastern Santigo, Chile.

Three food access profiles were confirmed (Figure 1). They were differentiated mainly by the place of food acquisition although some differences were also observed regarding food assistance components. The most prevalent profile (701 households, 70.2%) was called *Classic* (profile 1) because it included households that mainly obtained food in open markets, supermarkets, and neighborhood grocery stores. The second most prevalent profile (17.9%) was denominated *Multiple* (profile 2) because it was characterized by households that buy food in different outlets, including pharmacies. Finally, the Supermarket-Restaurant profile (11.9%) (profile 3) included households in which food was primarily purchased in a supermarket, which also had higher participation of restaurants compared to the other profiles. The first two profiles concentrated on government food support (>70% pre-pandemic and ~100% in lockdown), while in the third profile, coverage was much lower (15% pre-pandemic and 50% in lockdown).

The food access profiles were also related to sociodemographic variables (Table 2). Compared with the classic profile, households with a female head were more likely to be in the Multiple profile [RRR = 1.51; (CI: 0.99–2.32); *p*: 0.058]. Similarly, households with higher income and with higher maternal educational levels were more likely to be in the Supermarket-Restaurant profile [RRR = 12.30; (CI: 2.88–52.5); *p*: 0.001 and RRR = 4.58; (CI: 2.22–9.43); *p*: 0.000, respectively].

**Table 2:** External food access profiles by sociodemographic variables for 999 households in southeastern Santiago, Chile.

**Profile/variable**	RRR^*^	CI 95%^*^	*P*-value^*^
**Profile 1- Classic**	**Ref**.		
**Profile 2- Multiple**			
Income quintile	1.21	1.02–1.45	0.030
Female head of the household	1.51	0.99–2.32	0.058
Mother education	1.12	0.87–1.45	0.388
**Profile 3- Supermarket-Restaurant**			
Income quintile	12.30	2.86–52.58	0.001
Female head of the household	0.66	0.25–1.74	0.406
Mother education	4.58	2.22–9.43	0.000

* Multinomial regression gives relative risk ratio (RRR) and a *p*-value with 95% Confidence Interval (CI).

### 3.3. Food access profiles and child's dietary quality

We assessed the association between food access profiles and dietary quality (**Table 4**, unadjusted results). We observed that food access profiles were only associated with fish consumption (the Supermarket-Restaurant profile compared to the classic profile OR = 1.77; CI: 1.00–3.12; *p*: 0.048) and marginally with the overall compliance of DGA (Profile 3: coefficient = 0.17; CI: −0.04–0.39; *p*: 0.109).

### 3.4. Food access profiles and domestic environment characteristics

We also explored whether the domestic environment might influence these results. To do this, we first examined the association between the food access profiles and the domestic environment characteristics (food management and routine modification) (Table 3).

**Table 3:** Personal environment characteristics according to food access profiles.

	***n***	Profile 1-Classic %^*^	***P*-value**	**Profile 2, Multiple %**	***P*-value**	**Profile 3-Supermarket-Restaurant**	***P*-value**
**Set of food management variables**							
Woman buys food	995	83.4	Ref.	84.1	0.798	75.6	0.031
Woman prepares food	988	93.5	Ref.	92.0	0.440	85.7	0.007
Child has a meal frequency pattern	871	25.1	Ref.	23.9	0.771	34.0	0.061
Master ≥5 culinary skills	999	29.7	Ref.	48.6	0.000	47.1	0.000
**Set of routine modification variables**							
Increased online work hours, yes	942	22.2	Ref.	32.3	0.006	46.6	0.000
Increased domestic work hours, yes	984	54.3	Ref.	49.7	0.025	60.5	0.396
Reduction in person working hours, yes	934	31.9	Ref.	31.7	0.944	38.1	0.194
**Difficulty due to school closures**							
Making time for myself without the children	946	33.4	Ref.	31.5	0.642	36.3	0.530
Having a schedule for school activities at home	965	35.1	Ref.	38.7	0.395	36.4	0.781
Maintain communication with teachers	955	25.0	Ref.	25.6	0.812	22.6	0.564
Monitor the child's school activities	965	32.2	Ref.	31.2	0.730	31.0	0.754
Support the child's learning at home	965	28.8	Ref.	32.4	0.381	32.5	0.415

999 households in southeastern Santiago, Chile.

* Percentages are adjusted for sex and age of the child or adolescent. Univariate multinomial regressions were made between food access profiles and each personal environment variable, adjusted for sex and age of the child. The multinomial regressions provide risk relative ratio (RRR) along with their *p*-values and confidence intervals. RRRs were transformed to percentages for clarity.

In the set of food management characteristics, we found that most differences were concentrated between the Supermarket-Restaurant profile and the Classic one. The Supermarket-Restaurant profile concentrated on protective factors for children's diet quality: it was more frequent to find households with men in charge of preparing food (Profile 3: RRR = 2.11; CI:1.07–4.19; *p*: 0.032) and almost half of them had mastered a significant number of cooking skills [Profile 3: RRR = 2.16; (CI:1.44–3.25); p: 0.000]. The Multiple profile was only associated with mastering five or more culinary skills (Profile 2: RRR = 2.28; CI:1.63–3.21; *p*: 0.000).

In the set of routine modifications, we found that the Supermarket-Restaurant profile compared to the Classic one had a higher increase in online work during the pandemic [Profile 3: RRR = 3.25; (CI: 2.12–4.97); *p*: 0.000]. The Multiple profile also presented a significantly more increase in online work than the Classic profile [Profile 2: RRR = 1.79; (CI: 1.21–2.65); *p*: 0.004], but the increase in domestic work was significantly lower than in the Classic profile [Profile 2: RRR = 0.54; (CI: 0.33–0.89); *p*: 0.015], even after adjusting for other covariables. There were no significant differences between profiles concerning decreased in-person work and difficulties associated with school closures.

When adjusting the regressions by the food management variables retained, we did not observe changes in the results (Table 4). However, when adjusting the routine modification variables, we observed that in the Supermarket-Restaurant profile (i.e., the one whose routines were most impacted by the pandemic), the likelihood of compliance with the DGA (overall, fish, legumes, and dairy intake) increased. At the same time, no change was observed in compliance with fruit and vegetable intake and water consumption recommendations. As an example, the number of DGA recommendations accomplished increased from coefficient 0.17 (CI: −0.04 to 0.39; *p*: 0.109) in the univariate regression to coefficient 0.22 (CI: 0.00 to 0.44; *p*: 0.049) in the adjusted regression and the probability of consuming fish at least twice a week increased from OR = 1.77 (CI: −1.00 to 3.12; *p*: 0.048) to OR = 1.96 (CI: −1.09 to 3.52; *p*: 0.024). We did not observe significant changes in the Multiple profile associations when adjusting for domestic environment. No change was observed in UPF's intake.

**Table 4:** Association of food access profiles and children's dietary quality (*n* = 999 households in southeastern Santiago, Chile), crude and adjusted results.

**Ultra-processed foods daily intake**	**Univariate regression** ^*^			**Multivariate regression 1** ^**^ **(Adj. for food management)**			**Multivariate regression 2** ^***^ **(Adj. for routine modification)**		
	**OR**	**CI 95%**	***P*****-value**+	**OR**	**CI 95%**	***P*****-value**+	**OR**	**CI 95%**	***P*****-value**+
≥**5 ultra-processed foods**	**827 households**			**827 households**			**827 households**		
Classic profile	Ref.			Ref.			Ref.		
Multiple profile	1.15	0.80–1.66	0.442	1.12	0.77–1.62	0.556	1.14	0.80–1.65	0.477
Supermarket-Restaurant profile	0.86	0.56–1.32	0.494	0.84	0.54–1.29	0.421	0.88	0.57–1.37	0.582
**Dietary guidelines recommendations**	**Univariate regression** ^*^			**Multivariate regression 1** ^**^			**Multivariate regression 2** ^**^		
	**OR**	**CI 95%**	***P*****-value**+	**OR**	**CI 95%**	***P*****-value**+	**OR**	**CI 95%**	***P*****-value**+
**Number of recommendations accomplished**	**826 households**			**826 households**			**826 households**		
Classic profile	Ref.			Ref.			Ref.		
Multiple profile	0.02	−0.17–0.20	0.854++	0.01	−0.18–0.20	0.936++	0.03	−0.15–0.22	0.727++
Supermarket-Restaurant profile	0.17	−0.04–0.39	0.109++	0.16	−0.06–0.38	0.149++	0.22	0.00–0.44	0.049++
**Fish (**≥**2 weekly)**	**790 households**			**790 households**			**790 households**		
Classic profile	Ref.			Ref.			Ref.		
Multiple profile	1.12	0.63–2.00	0.692	1.09	0.61–1.97	0.762	1.14	0.64–2.04	0.655
Supermarket-Restaurant profile	1.77	1.00–3.12	0.048	1.73	0.97–3.07	0.063	1.96	1.09–3.52	0.024
**Legumes (**≥**2 weekly)**	**809 households**			**809 households**			**809 households**		
Classic profile	Ref.			Ref.			Ref.		
Multiple profile	0.74	0.49–1.11	0.141	0.70	0.46–1.06	0.091	0.76	0.50–1.14	0.184
Supermarket-Restaurant profile	1.10	0.69–1.73	0.693	1.03	0.65–1.64	0.898	1.16	0.73–1.85	0.527
**Dairy (**≥**3 daily)**	**818 households**			**818 households**			**818 households**		
Classic profile	Ref.			Ref.			Ref.		
Multiple profile	1.05	0.72–1.54	0.787	1.06	0.72–1.55	0.777	1.10	0.75–1.61	0.634
Supermarket-Restaurant profile	1.07	0.69–1.65	0.758	1.07	0.69–1.66	0.762	1.19	0.77–1.85	0.434
**Fruits and vegetables (**≥**5 daily)**	**822 households**			**822 households**			**822 households**		
Classic profile	Ref.			Ref.			Ref.		
Multiple profile	1.00	0.61–1.65	0.986	1.00	0.60–1.66	0.998	0.97	0.59–1.59	0.901
Supermarket-Restaurant profile	1.01	0.56–1.81	0.969	0.98	0.54–1.78	0.957	1.01	0.55–1.83	0.984
**Water (6 glasses daily)**	**787 households**			**787 households**			**787 households**		
Classic profile	Ref.			Ref.			Ref.		
Multiple profile	1.28	0.82–2.02	0.281	1.33	0.84–2.11	0.229	1.33	0.84–2.10	0.225
Supermarket-Restaurant profile	1.39	0.79–2.44	0.248	1.44	0.82–2.54	0.209	1.38	0.78–2.44	0.270

+Logistic regression gives Odds Ratio (OR) and a *p*-value with 95% of Confidence Interval (CI).

++Linear regression. Coefficient reported.

* Model adjusted for sex and age of the child.

** Model adjusted for sex and age of the child and food management variables retained in previous analyses (woman prepares food and master 5 or more culinary skills).

*** Model adjusted for sex and age of the child and routine modification variables retained in previous analyses (increase in online work hours and increase in domestic work hours).

## 4. Discussion

In a sample of middle- and low-income Chilean households with children living in similar external food environments, we could identify three food access profiles that vary depending on socioeconomic characteristics of the household, including gender of the head of the household. Household's food access profiles were poorly associated with the children's dietary quality, but the domestic environment influenced how food access related to the quality of dietary intake.

The Classic profile, the most prevalent profile in this sample, accounted for traditional methods of food access among low- and middle-income households in Chilean urban cities, including a combination of food purchases on open markets, supermarkets, and grocery stores, together with high access to government food transfers. However, we also found two other food access profiles in our sample.

The Multiple food access profile was characterized by households who buy foods in multiple food outlets, including small retail businesses, local shops, and even pharmacies. Other studies in Brazil have reported an increase in pharmacy food purchases (45) as a response to time optimization (46). During the COVID-19 lockdown, time away from home was significantly reduced (i.e., families were allowed two 1-h leave per week). Possibly, the main places of food purchases diversification were related to the need to optimize time by making less planned and quicker purchases. In fact, this profile was slightly more prevalent in households with lower income and a greater number of women heads in the household supporting diversification of outlets as a way of coping with less time-resource. Similar coping strategies have been described for Latina mothers who navigate less healthy environments (13).

Finally, the Supermarket-Restaurant profile was characterized by the purchase of delivery food. In Chile as in other Latin American countries, eating away has been increasing in the past decades although the quality of the food purchased varies significantly depending on the socioeconomic classes. In Brazil, it has been reported that people of the highest income levels present the highest expenditure on out-of-home food (47). During the COVID-19 lockdown, there is evidence that families from higher educational levels and higher income were the ones that most frequently used digital channels to access food (48). In Chile, people buy time by buying ready-to-eat food or paying for a maid. Households that cannot afford to pay for food-time experienced more stress. Correspondingly, in this study, we observed that this profile was more frequent among families with higher education and income. Moreover, households in this profile were not part of food assistance programs before the pandemic, but their coverage increased given the difficulties created by the pandemic.

Our study also shows that the profiles were poorly related to the quality of the children's dietary intake. This is likely because this was a relatively homogeneous urban population, in which food availability in the external environment was assured. We observed that almost half of the sample consumed five or more UPFs daily, independently of the type of store where the profile accessed food. In the Brazilian case, Machado et al. (49), reported a positive association between purchasing foods at supermarkets and UPF consumption. However, in our results, the Supermarket-Restaurant profile was not associated with a higher UPF intake in these children. We believe these findings suggest that UPFs have penetrated all types of food stores including open markets. This is aligned with the results of Spires et al. (50) who studied three countries of high-, middle-, and low-income groups, showing that sweetened beverages and confectionaries had permeated all food outlets, independent of the country's income level. Food swamps—urban spaces with high-density unhealthy foods—are becoming a significant concern in countries facing post-transitional nutrition transition phases such as Chile and several Latin-American countries (51).

Overall, in our sample, consumption of healthy foods was low. Approximately 10% followed three or more DGA recommendations (52). This is in line with previous studies that have shown high consumption of UPF by Chilean adults and children and low consumption of healthy foods (18). This is also concordant with advanced stages of the nutrition transition in which increased industrialization of food systems, technological changes, globalization, and transnational food industry penetration has promoted a diet dominated by UPF that displaces the consumption of healthy foods, worldwide (53). Chile has recently implemented the Food Labeling and Advertising Law that considers multiple mutually reinforcing policies including the use of front-of-package warning labels, restricting child-directed marketing, and banning school sales of unhealthy foods to counteract the increase in UPF consumption (54). Initial data showed significant results (55–57); however, the fact that the consumption of UPF continues to be very high indicates the need of implementing complementary policies, also including the promotion of healthy food consumption. Furthermore, reports indicate that Chile had the worst dietary quality during the COVID-19 quarantine among 11 LAC countries (58). A recent evaluation of the Food Labeling and Advertisement Chilean Law reported that mothers from lower socioeconomic status found healthier foods financially inaccessible (59). Several studies have shown that a quality diet is more expensive than one based on UPF (60, 61). In the case of Chile, Cuadrado and García established in 2015 that a food basket that complies with DGA recommendations is 36.1% more expensive than a basic food basket (62). This implies that we must ensure food accessibility in terms of affordability. In the context of the global food crises, this will increasingly become a challenge for lower-income families in most countries (63).

We also demonstrated that the domestic environment's variables, such as changes in routine, could influence the impact of food access on the quality of children's diets. Mastering different culinary skills and having a meal structure have been described as protective factors of the children's diet (35, 36). Several studies from high-income countries have reported that these variables were reinforced by the parent's obligation to stay home during the lockdown as they had more time for food chores (64–66). Our results suggest the contrary since the effect of time disruption on the quality of children's intake was more important than the protective factors. This finding aligns with Jansen et al. (39) study in the US. They reported that households with children did not experience the diet-beneficial effects of the lockdown due to the increased stress levels experienced by parents (caused by the disruption of habits and school closures). On the contrary, the pressure was associated with a worsening of the children's intake.

Our results confirmed that the disruption of timing in domestic environments—produced by COVID-19—impaired the quality of children's diets. Time constraints have been described as a barrier to home cooking in the US (67), Ireland (68), and in general, in high-income countries (69). More evidence is needed in the context of middle-income countries. However, a study conducted in 10 low- and middle-income countries regarding the food and financial crisis in 2007 showed that time constraints could worsen dietary intake (70). A recent study conducted in adolescents shows that convenience emerges as a relevant determinant of food choices when food availability is no longer an issue (71). Our study shows that addressing time issues is needed to better understand barriers to healthy eating at the household level. Studies that can dive deeper into household dynamics might give us some clues on how to materialize these interventions (72).

Moreover, time in the domestic food environment is women's time. Worldwide and in Chile, mothers are the food gatekeepers. i.e., those responsible for the entire food cycle in the household (73, 74). Our results showed that women are almost exclusively responsible for buying and preparing food in all the external food access profiles. According to Clark et al. (75), the pandemic has had a more significant impact on women and has changed household dynamics. As a result, mothers are now taking on more and disproportionately caregiving responsibilities. In Australia, Craig and Churchill (76) reported that although both parents were forced to stay home, gender gaps in housework and care increased. Interestingly, we observed a higher share of this food gatekeeper role in the food access profile that seemed to have better dietary quality (i.e., Supermarket-Restaurant profile). This could indicate that incorporating more people in the household food cycle, i.e., more “personal times,” is a protective factor for the quality of the children's diet. As Constantinides et al. (77) established, it is crucial to incorporate gender equity in the food environment framework as their dynamics in the domestic environment affect the entire food cycle, primarily through time use (78, 79).

Remarkably, in the profile that gathered the women head of household (the Multiple one), although online work during the pandemic increased, the rise in domestic work was the smallest among the three profiles. These results suggest, on the one hand, that women heads of household have access to jobs that did not allow them to stay at home (informal jobs or jobs considered essential such as supermarket cashiers and stocking shelves, among others) (80). On the other hand, women's domestic responsibilities did not increase in this group because they were already very high even under normal pre-pandemic conditions (48). Notably, the quality of children's diet in this profile was not significantly lower than that of the other profiles. Evidence shows that these households were the most affected during the pandemic, particularly those of low income (81). Probably, women implement coping strategies, such as preparing meals in advance and using convenience foods (79), that allow them to maintain the dietary quality of their children even under disruptive conditions. These strategies are really the response to “juggling responsibilities,” and they might have negative implications for women's own health (79). Thus, incorporating a gender perspective into policies promoting healthier nutrition is currently imperative.

## 5. Limitations of the study

This study has several limitations that are worth mentioning. First, our self-reported dietary intake measurement could introduce potential biases, and using DGA for assessing dietary quality is limited. However, these methods are those traditionally used and can give us a fair idea of children's dietary quality. Second, in the latent class analyses, the names of the profiles are assigned by the researchers, which can lead to a “naming fallacy.” In this study, we named the profiles primarily according to their highest probability of food access and secondarily by highlighting their differences. Third, using a survey approach to the domestic environment is partial; qualitative research could help provide a deeper understanding of the role of the domestic environment. Fourth, the representativeness of this study is restricted to an urban area where food availability is assured. Moreover, we worked only with lower-middle socioeconomic households, but socioeconomic status directly influences diet quality. Further research, including households of other contexts, such as rural sectors and higher socioeconomic status, could improve our understanding of the external and domestic environment interactions in the children's dietary intake quality.

## 6. Conclusion

We found that food access was poorly related to the dietary quality of low-middle-income Chilean children and adolescents, highlighting that in this urban context, promoting healthier food environments requires interventions that restrict access to UPF foods and favor the affordability of fresh foods rather than just focusing on their availability. In the context of economic crisis and food price inflation, these measures will be particularly relevant for vulnerable groups. Moreover, our results suggest that to improve dietary quality, we also need to intervene intra-households' dynamics and roles that affect food access. Currently, the concentration of the food gatekeeper role on women and their lack of time to fulfill all their responsibilities is a risk factor for children's nutrition and for women's health. Thus, incorporating a gender perspective into nutritional interventions should be a priority to advance in ensuring better diets for all.

## Data availability statement

The original contributions presented in the study are included in the article/supplementary material, further inquiries can be directed to the corresponding author.

## Ethics statement

The studies involving human participants were reviewed and approved by Comité de Ética de Investigación en Seres Humanos- Universidad de Chile - Facultad de Medicina. Written informed consent to participate in this study was provided by the participants' legal guardian/next of kin.

## Author contributions

IP and CC conceptualize the study. CC provided essential materials and secure funding for the study. IP wrote the first draft of the manuscript. IP and PF conducted data analyses. CC, IP, FM, and MG contributed to the interpretation of the results. All authors contributed to the manuscript writing and read and approved the final manuscript.

## Footnotes

^1^The minimum wage in Chile to date was USD 400 aprox.
